# Prognostic role of CD44 expression in osteosarcoma: evidence from six studies

**DOI:** 10.1186/1746-1596-9-140

**Published:** 2014-08-11

**Authors:** Yu Liu, Yongwei Wu, Sanjun Gu, Zhenzhong Sun, Yongjun Rui, Jianbing Wang, Yao Lu, Haifeng Li, Kelin Xu, Peng Sheng

**Affiliations:** Department of Orthopedics, The Ninth People’s Hospital of Wuxi City, The Affiliated Wuxi Hospital of Soochow University, No.999 Liangxi Road, Wuxi, Jiangsu, Province 214062 China

**Keywords:** CD44, Bone tumor, Osteosarcoma, Prognosis, Meta-analysis

## Abstract

**Background:**

Numerous studies examining the relationship between CD44 expression and prognostic impact in patients with osteosarcoma have yielded inconclusive results. The aim of this meta-analysis was carried out to investigate the relationship between CD44 expression and the survival in patients with osteosarcoma.

**Methods:**

We therefore conducted a meta-analysis to provide a comprehensive evaluation of the prognostic role of CD44 expression on the overall survival rate and metastasis, which compared the positive and negative expression of CD44 in patients of the available studies.

**Results:**

A detailed search was made in MEDLINE and EMBASE for relevant original articles published in English. Finally, a total of six studies with 329 osteosarcoma patients were involved to estimate the relationship between CD44 expression and metastasis of tumor and overall survival. Positive expressions of CD44 did not predict neoplasm metastasis (RR = 1.36, 95% CI: 1.00–1.84, P = 0.50), and the results indicated that higher expression of CD44 could not predict poorer survival in osteosarcoma with the pooled HR of 0.55 (95% CI: 0.27–1.13, P = 0.47).

**Conclusions:**

The findings from this present meta-analysis suggest that CD44 expression is not associated with overall survival rate and metastasis in osteosarcoma.

**Virtual Slides:**

The virtual slide(s) for this article can be found here: http://www.diagnosticpathology.diagnomx.eu/vs/1373995521295618

## Background

Osteosarcoma is the most common malignant primary bone tumor and the majority of these tumors occur among children and adolescents [1–3]. Despite the development of neoadjuvant chemotherapy, the 5-year survival rate for patients with high-grade osteosarcoma is still less than 50% [4]. The prognostic factors that have been implicated include demographics (age and sex), tumor size, site, stage, and response to chemotherapy. However, the mechanism of prognosis in osteosarcoma patients is still not fully understood. Therefore, a better understanding into its basic biology is urgently needed to identify its prognostic markers and therapeutic targets [5, 6]. In recent years, the expression of certain biological molecules has been identified as potential prognostic markers for osteosarcoma, including the expression of CD44.

CD44 is the major hyaluronan (HA) receptor [7], and CD44 bound to HA has been proven to participate in various tumor biological activities, including tumor progression, metastasis and proliferation [8, 9]. Some variant isoforms of CD44 (CD44V) are reportedly associated with increased invasion, metastasis, and poor prognosis [10]. It has been reported that CD44V6 can regulate the extracellular matrix, promote cell motility, and suppress tumor apoptosis. In fact, CD44V6 has been implicated in promoting tumor progression [11]. CD44 proteins have been studied in relation to tumor malignancy and metastatic potential. The prognostic value of CD44 for patients with cancer has been reported in various solid tumors, including colon, lung, and breast cancer [12–14]. With respect to osteosarcoma, the relationship between CD44 expression and prognosis was still controversial [15, 16].

Most of earlier studies suggested CD44 high expression was associated with high risk of tumor metastasis and worse survival in patients with osteosarcoma. However, some other studies showed insignificant or opposite results. Therefore, the aim of this meta-analysis was carried out to investigate the relationship between CD44 expression and the survival in patients with osteosarcoma. We also discuss the possibility of using CD44 as a prognostic marker in osteosarcoma.

## Methods

### Search strategy

The PubMed, EMBASE, and MEDLINE databases were searched, in addition to the Cochrane Central Register of Controlled Trials, to locate articles (published between January 1994 and January 2014), including articles referenced in the publications. The search strategy included the following keywords variably combined by “CD44”, “osteosarcoma”, “bone tumor” and “prognosis”. Internet search engines were also used to perform a manual search for abstracts from international meetings, which were then downloaded and studied.

### Inclusion and exclusion criteria

Studies met the inclusion criteria if they studied the patients with osteosarcoma, measured the expression of CD44 in cancer tissue and investigated the association betweenCD44 expression levels and survival out come. When a study reporting the same patient cohort was included in several publications, only the most recent or complete study was selected. Studies of case reports, letters, and reviews without original data; non-English papers; animal or laboratory studies; and studies of nondichotomousCD44 expression levels and absence of survival outcome were excluded. If any doubt of suitability remained after the abstract was examined, the full manuscript was obtained [17].

### Data extraction

Two review authors assessed the methodological quality of potentially eligible studies, without consideration of the results. Extracted data were then crosschecked between the two authors to rule out any discrepancy. Data regarding the following for each included studies were extracted independently: first authors’ surname, publication year, origin country, sample size, CD44 assessment methods and the cut-off definition, and HR of CD44 expression for overall survival (OS) as well as corresponding 95% confidential interval (CI) and P value. Multivariate Cox hazard regression analysis reported in the article was included in the present analysis. Disagreements were discussed by the authors and resolved by consensus.

### Statistical analysis

The statistical analysis was carried out using the Review Manager (RevMan) software version 5.0(The Nordic Cochrane Centre, The Cochrane Collaboration, Copenhagen, Denmark). All these HRs and 95% confidence interval(CI) were calculated following Tierney’s method. Pooled HR was calculated using a fixed-effects model or random-effects model to evaluate the relationship between CD44expression and overall survival. I^2^ statisticswas used to evaluate the between-study heterogeneity analysis in this meta-analysis [18]. The random effects model was used when an obvious heterogeneity was observed among the included studies (I^2^ > 50%). The fixed effects model was used when there was no significant heterogeneity between the included studies (I^2^ ≤ 50%). Publication bias was estimated using a funnel plot with an Egger’s line arregression test; funnel plot asymmetry on the natural logarithm scale of the HR was measured by a line arregression approach.

## Results

### Eligible studies

**Figure 1:**
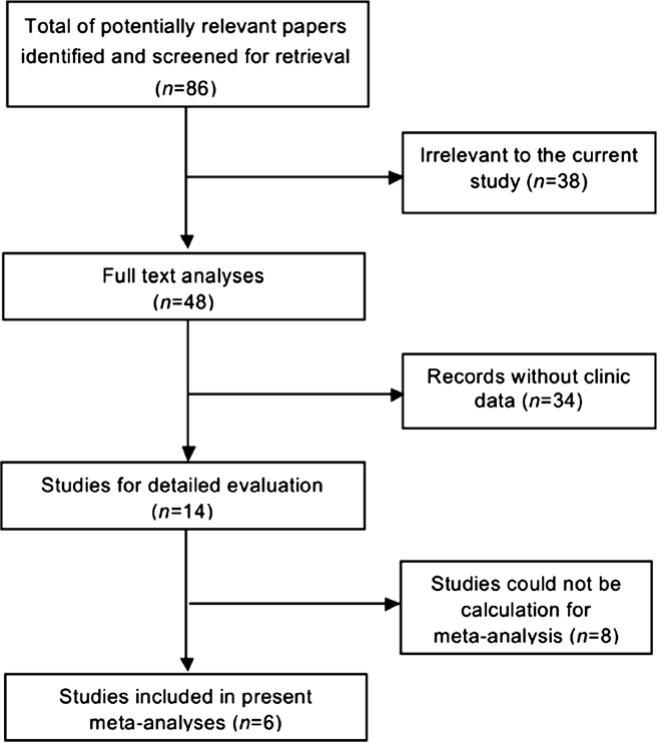
**Flow diagram of the study selection process.**

The initial search retrieved a total of 86 references, and after screening titles and abstracts of identified articles, 38 were excluded because they were not related to the current study. Upon further review, 34 were excluded because they were either laboratory studies or records without survival data. Then we evaluated 14 potential candidate studies in full text. It was found that the survival data of one article could not be used in this study. Finally, 6 [19–24] studies were included in this meta-analysis, which were published between1994 and 2014 (Figure 1). All of them were retrospective in design. In each study, the cut-off values of CD44 appeared to be different. The main characteristics of the included studies were summarized in Table 1.

**Table 1:** **Characteristics of studies included in the meta-analysis**

						Expression of CD44 (n)		OS/RFS (%)		Metastasis (n)	
Study	Year	Age (median)	No. of patients	Method	CD44 cut-off	Positive	Negative	CD44 (+)	CD44 (-)	CD44 (+)	CD44 (-)
Boldrini et al. [19]	2010	15.9	34	IHC	≥10%	17	17	21.5%	25.3%	NA	NA
Deng et al. [20]	2013	18.3	90	IHC	≥25%	59	31	NA	NA	38	12
Gvozdenovic et al. [21]	2013	NA	53	IHC	NA	9	44	NA	NA	4	15
Kim et al. [22]	2002	17	50	IHC	≥50%	10	40	NA	NA	4	22
Kuryu et al. [23]	1999	19	39	IHC	≥10%	18	21	24%	58%	13	10
Ma et al. [24]	2011	16	63	IHC	NA	62	1	40.3%	0%	12	0

### Meta-analysis

**Figure 2:**
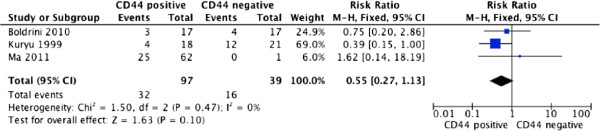
**CD44 expression and overall survival rate of osteosarcoma patients.**

**Figure 3:**
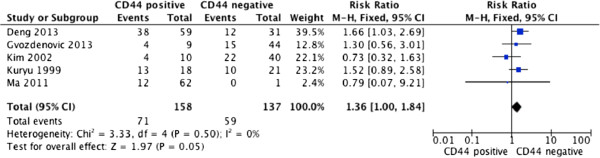
**CD44 expression and metastasis of osteosarcoma patients.**

For studies evaluating overall survival (OS), there was no between-study heterogeneity among those six studies for CD44 (*I*^2^ = 0%), so the fixed-effect model was used to calculate the pooled HR with corresponding 95% CI. The result indicated that positive expressions of CD44 did not predict neoplasm metastasis (RR = 1.36, 95% CI: 1.00–1.84, P = 0.50), and higher expression of CD44 could not predict poorer survival in osteosarcoma with the pooled HR of 0.55 (95% CI: 0.27–1.13, P = 0.47). (Figures 2, 3)

### Publication bias

Funnel plot and Egger’s test were used to evaluate the publication bias of the literatures. The shape of the funnel plot did not reveal any evidence of obvious asymmetry (Figures not shown).

## Discussion

Osteosarcoma is a life-threatening bone malignancy that often occurs in teenagers. It is the second leading cause of cancer-related death in pediatric age group and young adults [25]. Current treatments for osteosarcoma include surgical resection of both primary and pulmonary lesions, chemotherapy, and radiotherapy. Disease-free survival escalated from <20% prior to the introduction of effective chemotherapy to around 60% and overall survival to 60–70% [26]. At present, the ability to predict the prognosis of osteosarcomas is limited. Therefore, identifying prognostic markers of survival in osteosarcomas could be informative for selecting proper management. Traditional prognostic markers, such as gender, age, tumor location, disease-free interval, tumor doubling time, representation, and number of detectable pulmonary metastases, have had limited success in identifying those patients that need aggressive chemotherapy and those that do not [27]. In recent years, a number of cell surface markers were found to indicate a small group of cancer cells, referred as cancer stem cells, which are responsible for tumor initiation, progression, metastasis and drug resistance [28]. Many researchers have reported that high expression of these markers indicates bad clinical features and poor prognosis [29, 30], and CD44 was one of the most reported cancer stem cells markers.

CD44 was previously thought to be a transmembrane adhesion molecule, which also played a role in the metabolism of its principal ligand hyaluronan. It may exist in three distinct physical phases, as a transmembrane cell surface receptor, an integral component of the matrix and in a fluid phase, each with the potential for being functionally significant. CD44 is known to be a major hyaluronic acid receptor in vitro, although it is not known how often CD44 is expressed or what role it plays in normal bone tissues. CD44 proteins have been observed in osteoclasts and osteocytes by means of immunohistochemical analysis on bone tissue, but there have been some controversial reports that CD44 proteins were found in osteoblasts [31]. As CD44 reacts with the extracellular matrix, several published reports have suggested that CD44 expression is related to metastatic potential, prognosis, and the biologic properties of human malignancies. Investigations of CD44 over the past 20 years have established additional functions for CD44, including its capacity to mediate inflammatory cell function, tumor growth, adhesion, migration and metastasis. It has also become evident that intricate post-translational modifications of CD44 regulate the affinity of the receptor for its ligands [32]. Whether CD44 is a prognostic marker in osteosarcoma patients has been studied extensively, but the conclusions are inconsistent. This meta-analysis was carried out by critically reviewing six studies on the association of CD44 with prognosis in osteosarcoma.

The present meta-analysis showed that high CD44 expression did not indeed predict poor survival and metastasis in patients with osteosarcoma. However, it should be circumspect to make a verdict of the association with CD44 and osteosarcoma, because there are still several issues should be considered. First, since the number of included studies in this meta-analysis was only six, it might weaken the reliability of our results. More well-designed clinical studies with large cases of osteosarcoma should be performed in the future to validate the relationship between CD44 expression level and prognosis of osteosarcoma patients. Second, lack of abundant CD44 expression data in global population makes it difficult to set a standard value for the measurement of CD44. Third, the methods used for the evaluation of the levels of markers in osteosarcoma patients and the use of standard threshold, are both likely to impact on our results. Although immunohistochemistry was the most commonly applied method, the cut-off value was defined differently in inclusion studies. Therefore, we strongly suggest conducting more prognostic studies for high CD44 expression in osteosarcoma.

Moreover, recently studies have demonstrated that microRNAs might influence chemoresistance of osteosarcomas with different pathways including CD44, resistance to chemotherapeutic agents is still one of the major reasons for the failure of osteosarcoma treatment, while we did not excluded the role of CD44 in chemoresistance of osteosarcoma. The identification of cancer-specific miRNAs and their targets is pivotal for understanding their role in tumorigenesis and metastasis, and may be important for the discovery of novel therapeutic targets. CD44 which contained the corresponding binding site of microRNAs’ 3′UTR, was regulated by some microRNAs, such as miR-34a, miR-140 and miR-215 [33–35]. The results of these published studies showed that the CD44 level inversely correlated with the microRNAs level. So in the future research, we will discuss the role of CD44 in chemoresistance of osteosarcoma.

On the other hand, many researchers have reported that high expression of some markers indicates bad clinical features. Such as clinical stage, positive distant metastasis and poor response to chemotherapy [36, 37]. In this present study, we also discussed the relationship between overexpression of CD44 and clinicopathological parameters in osteosarcoma patients. Nonetheless, the result showed that no significant difference was observed between the expression of CD44 and patients’ age, gender, tumor size, clinical stage, positive distant metastasis and poor response to chemotherapy (data not shown).

Despite the inherent limitations of meta-analysis on prognostic literature, this meta-analysis, representing a quantified synthesis of all published studies of CD44, has shown that the high expressed CD44 is not significantly associated with poor survival and metastasis in patients with osteosarcoma. For better analysis the relationship between CD44 expression and prognostic with the osteosarcoma, it is necessary to improve the experimental methods and detection methods, and to clear a unified quantitative standard. Future adequately multi-center designed prospective with larger sample size were of great value to confirm these findings and more clinical studies should be carried out before the application of CD44 in prognosis of osteosarcoma.

## Conclusions

The findings from this present meta-analysis suggest that CD44 expression is not associated with overall survival rate and metastasis in osteosarcoma. CD44 may not be a useful marker to predict prognosis of osteosarcoma.

## Authors’ information

Yu Liu and Yongwei Wu: co-first authors.
